# Female Entertainment Workers and Condom Use Negotiation in Post-100% Condom Use Era Cambodia

**DOI:** 10.1007/s10508-020-01649-3

**Published:** 2020-03-24

**Authors:** Carinne Brody, Rebecca Reno, Pheak Chhoun, Kathryn Kaplan, Sovannary Tuot, Siyan Yi

**Affiliations:** 1 Public Health Program, College of Education and Health Sciences, Touro University California, Vallejo, CA 94592 USA; 2 School of Public Health, University of California, Berkeley, CA USA; 3 KHANA Center for Population Health Research, Phnom Penh, Cambodia; 4 Saw Swee Hock School of Public Health, National University of Singapore and National University Health System, Singapore, Singapore

**Keywords:** Condom use, Female entertainment workers, Female sex workers, HIV, Condomless sex, Focus groups

## Abstract

Beyond the monopoly environment of the early 100% Condom Use Program in Cambodia, less is known about how current female entertainment workers negotiate condom use on their own, and what factors impact that negotiation. This study aims to understand the experiences of current female entertainment workers in negotiating condom use with clients in Cambodia. Data collection occurred over a period of 3 weeks (August–September 2017) with concurrent data transcription and translation. A total of 27 focus group discussions (FGDs) were conducted in the following groups: pilot FGD (5), karaoke bar (5), massage parlor (5), beer garden (5), on-call sex workers (3), cross-venue groups exploring parenting issues (2), and street-based sex workers (2). Female entertainment workers experience a range of control over negotiating condom use with clients. Participants reported times when they were able to take direct action and successfully insist on condom use, times when they agreed to participate in condomless sex for money in the face of economic insecurity, and times when male clients sabotaged their attempts to negotiate condom use with tricks, verbal threats or threats of violence. These experiences are influenced by alcohol use, economic shocks, trust between partners, and experiences with side effects. Our findings support the development of policies that re-invigorate the structural-level condom promotion programs while also acknowledging the many individual-level factors that shape condom use such as alcohol consumption, economic insecurity, trust, and side effects.

## Introduction

The prevalence of HIV in Cambodia continues to be among the highest in Asia despite successful prevention efforts that have reduced the prevalence of HIV among the general public. The regional average among adults aged 15–49 is 0.2% compared to 0.5% in Cambodia (Joint United Nations Programme on HIV/AIDS, 2012). Cambodia’s HIV epidemic is fueled primarily by heterosexual transmission between female sex workers and male clients. According to the results of Cambodia National Center for HIV/AIDS, Dermatology and STD, the prevalence of HIV among female entertainment workers (FEWs) decreased from 4.6% in 2010 to 3.2% in 2016 and condom use with the most recent clients was at 91.8% in 2016 (National Center for HIV/AIDS and Dermatology and STD, 2017).

FEWs in Cambodia, who work at entertainment venues such as karaoke bars and massage parlors and may engage in transactional sex, face triple vulnerabilities in terms of their control over condom use with their clients (Rojanapithayakorn & Phalla, 2000). They are female in a male-dominated society; they are financially insecure in a low-income country and their work is illegal. Their ability to negotiate condom use on their own is extremely limited. They face clients that insist on condomless sex by offering more money or threatening violence (Dalla, Xia, & Kennedy, 2003; Tan & Melendez-Torres, 2016). Individual-level interventions that aim to build sex workers capacity to negotiate condom use have been found to be largely ineffective because of the extreme vulnerability and the low negotiation position of female sex workers (Hong, Fang, Li, Liu, & Li, 2008; World Health Organization, 2004).

In many countries throughout Asia, a nationwide structural-level program called the 100% Condom Use Program (100% CUP) has been largely successful in increasing condom use between sex workers and clients (World Health Organization, 2004; Zhongdan et al., 2008; Zou, Xue, Wang, & Lu, 2011). The central tenet of the program is the cooperation of the highest local authorities within the health services, law enforcement, local government and all managers of sex work establishments such as brothels. This top-down enforcement within all sex work establishments creates a “monopoly environment” within which clients have no choice but to use a condom because all establishments have the same rules (Rojanapithayakorn & Phalla, 2000).

During the generalized HIV epidemic in Cambodia (1990s), an evaluation of a national individual-level program that consisted of outreach workers providing educational materials about HIV and training in condom use negotiation showed that reported condom use was increasing moderately, but HIV infection rates among sex workers had risen to 57% (WHO, 2004). When Thailand adopted 100% CUP and was seeing early success, Cambodian officials followed suit and quickly scaled the program nationwide. The 100% CUP program is said to be responsible for the rapid decline of HIV in the nation; the Cambodia government was recognized by the United Nations for these efforts that contributed to a decline in the prevalence of HIV from an estimated 2.0% among the general population in 1998 to 0.8% in 2008 (Joint United Nations Programme on HIV/AIDS, 2010).

As the 100% CUP program has continued, evidence suggests that the results may have waned partially due to the changing sex work policy environment in Cambodia. In 2008, Cambodia implemented a law to ban brothel-based sex work—the Law on Suppression of Human Trafficking and Sexual Exploitation (Pearshouse, 2008). The enforcement of this “brothel ban” may be creating a cascade of unintended negative consequences impacting condom use (Joint United Nations Programme on HIV/AIDS, 2012). Studies have shown that new sex workers are less likely to wear condoms and more likely to have sexually transmitted infections (STIs), which suggests that the program may be lacking the capacity to monitor and evaluate the program for continued improvement (Sopheab, Morineau, Neal, Saphonn, & Fylkesnes, 2008). For example, since this law was passed, there have been frequent raids on establishments, such as entertainment venues, where sex is allegedly being sold and the presence of condoms is being used as evidence of sex work (Sarom, 2019; Soumy & Kohlbacher, 2018). Even carrying condoms can be a cause for harassment by police (Human Rights Watch, 2010; Maher et al., 2011). In addition, those who sell sex are left to negotiate condom use from an even more vulnerable position with the criminalization of sex work, particularly without security from the establishment where they work (Wong et al., 2018). As a result, evidence suggests that condom use between male clients and female sex workers has declined even more (Maher et al., 2011; Wong et al., 2018).

The changes in the structural environment within which condom use is happening has been documented, but we do not have documentation of the experiences of current FEWs as they negotiate condom use in this new environment. Without the monopoly environment of the early 100% CUP era, little is known about how FEWs navigate condom use with clients and what factors impact that negotiation. This study aims to understand the experiences of FEWs in negotiating condom use with clients in Cambodia.

## Method

This qualitative study was conducted as part of the development of the Mobile Link project, an operational mobile health research project coordinated by KHANA, the largest national HIV organization in Cambodia and their academic partner, Touro University California Public Health Program, which aims to engage FEWs through frequent automated voice messages (VMs) or text messages that link them to existing prevention, care and treatment services in the country. The 60-week randomized controlled trial is underway to evaluate the Mobile Link intervention. More details about the Mobile Link project can be found in the published protocol (Brody et al., 2019).

Intervention development consisted of a rigorous formative study that aimed to understand priority health issues among FEWs in order to tailor the intervention to the current FEW populations. In four provinces in Cambodia, focus group discussions (FGDs), in-depth interviews and data validation workshops where participants responded to sample VM and text messages were conducted to inform the Mobile Link message creation and program implementation. This study is based exclusively on the 27 FGD transcripts.

FGD methodology was used to explore participants’ lived experiences and reflections on those experiences including “how they think and why they think that way” (Kitzinger, 1995). We chose to conduct FGDs in order to understand the full range of experiences of our participants including their shared and unique reflections on how and why they think that way. There are some methodological limitations related to the use of a FGD design, for example it can advance more normative discourses, particularly related to the discussions of sensitive topics (Smithson, 2010). Despite these limitations, the study team decided that the benefits of this study design, including the ability to engage more participants in a cost-effectively and timely manner, outweighed the limitations.

### Procedure

Due to the intention to reach the most-at-risk population, the research team purposively selected to conduct the FGDs in the three provinces with the highest HIV prevalence (Phnom Penh, Battambang, and Banteay Meanchey), as well as Siem Reap, which has been identified as a high-burden area despite having a lower HIV prevalence. The formative research was implemented in partnership with community-based organizations: KHEMARA and Save Incapacity Teenager in Phnom Penh, Cambodian Women for Peace and Development in Battambang and Siem Reap and Partners for Development in Banteay Meanchey.

A two-stage randomized cluster design was used to recruit participants for the research. The research staff from KHANA, the largest national HIV organization, used a list of venues from a national entertainment venue mapping project to randomly select as many venues as possible (up to eight in total) within each venue type (karaoke bars, massage parlors, restaurants/beer gardens), within each province. Street-based and freelance sex workers were also included in some provinces. Next, outreach workers used convenience sampling to identify one FEW at each randomly selected venue.

A uniform screening tool was utilized to identify if selected participants were eligible. Eligibility requirements for participation in all FGDs were working at an entertainment venue in Cambodia or self-identified as a FEW, being in the age group of 18–30 years old, being currently sexually active, defined as having engaged in oral, vaginal or anal sex in the past 3 months, owning a personal and private mobile phone, knowing how to retrieve VM or retrieve and read SMS on a mobile phone, and being able to provide informed consent. Participants were offered $5 USD cash stipend for their participation.

Data collection occurred over a period of 3 weeks (August–September 2017) with concurrent data transcription and translation. A total of 27 FGDs were conducted in the following groups: pilot FGDs (5), karaoke bar (5), massage parlor (5), beer garden (5), on-call sex workers (3), cross-venue groups exploring parenting issues (2), and street-based (2). On average, participants were 26 years old. The FGDs were distributed evenly across geographic sites to ensure fair representation of geographic-specific information. The project coordinator oversaw the implementation of the FGDs, as well as coordination of transcribers and translators.

After each FGD was completed, the data collectors completed an FGD summary form together, which provided contextual and subjective information related to the FGDs to inform the analyses. All FGDs were audio recorded. After each FGD, all audio files were transferred from the digital recorder to a locked folder on the data collector’s laptop. Transcribers transcribed the data in Khmer. Transcribers were instructed not to improvise or assume meaning at all. If they could not understand a word, then they could write [unintelligible]. If they could not understand a large section, they sought the support of the project coordinator. Translators then accessed the Khmer transcripts to translate into English for content analyses. Translators were instructed to translate exactly as the transcription was written and if a phrase did not directly translate, they were asked to keep the phrase in Khmer and include in brackets a general meaning of that phrase.

### Measure

A FGD guide was developed that asked participants about a range of sexual and reproductive health topics including condom use. The FGD guide was pilot tested in each study province with a group of 8–10 women recruited predominantly from karaoke bars. A team of seven data collectors and five community lay health workers underwent a 2-day workshop on how to use the FGD guide, and basics of conducting FGDs around sensitive topics. During the tool development and pilot testing activities, the project team discussed the recruitment strategy and the FGD guide which influenced the final version of the FGD guide. The data from the pilot FGDs were integrated in the results of the larger study, but the participants who were part of the pilot FGDs did not participate in a further FGD.

Based on studies exploring condom use among FEWs in Cambodia and neighboring countries, the team developed six questions on condom use. For this population, we have found that it can be challenging to generate discussions during FGDs. We have found that asking some closed-ended questions first can allow participants to warm up to participation in discussions. Our FGD guide reflects this by listing closed-ended questions followed by open-ended questions for most topics. The purpose of collecting information was explicitly to develop messages for the Mobile Link program, so the questions were developed for that purpose, rather than to directly explore the experiences of negotiating condom use in entertainment venues among FEWs. The questions and initial probes for condom use were:

1. Think about the different sexual partners you have in your life: husband, sweetheart, client, maybe others… how would you describe condom use with those different people (Prompts: Who do you use them with, how often? Why some and not others? For those who you don’t use condoms with, why not?)
2. How much do you feel like you have a choice, or the power to decide about condom use?
3. Is it hard or easy to negotiate condom use? What are the situations when it is hardest to negotiate condoms?
4. Does your husband or sweetheart think they are your only partner? What would happen if they knew you had clients?
5. If you had an expert, like a doctor or nurse here with you, and could ask them questions about condoms, condom use or lubricant, is there anything you would want to know?

### Data Analysis

Qualitative data were analyzed using Dedoose (Dedoose qualitative coding software, Version 8.0.35, 2018). All 27 FGD transcripts were uploaded into the software. Two researchers independently labeled sections of transcripts related to the six condom use questions and created initial codes. The researchers came together to discuss initial codes and to group them into emerging themes. This process of coding continued iteratively until a final codebook was created. The codebook was refined several times and from the final codebook, a diagram that summarized the conceptual relationships between the codes was created. The conceptual model was influenced by existing models from complimentary literature such as condom use self-efficacy among sex workers (Wang et al., 2009) and the continuum of volition (Weissman et al., 2006). Example quotes that demonstrate each theme are displayed below and identified by their participant number, city of data collection and FGD number. When a participant number could not be identified because the audio recording was not clear enough, we used the identification “Participant X.”

All key research and data collection personnel involved in this study completed the online the National Institute of Health research ethics course on the protection of human research participants. All FGD participants were informed of the study purpose and the risks and benefits to their voluntary participation. The data collectors read the informed consent to the participants as it was written. Each participant was given two copies of the consent form, translated into Khmer. The data collectors ensured that the FGD space was quiet and confidential. If the data collector was not satisfied with the level of privacy or quietness, she was instructed to identify a more suitable space. Facilitators were told that it was better to reschedule than to compromise the privacy of the participants.

The study topics included personal information about extremely sensitive topics. All participants were offered escorted referrals to counselors and health services upon request, including to a local women’s center if women had experienced, or were experiencing, intimate partner violence.

## Results

**Fig. 1:**
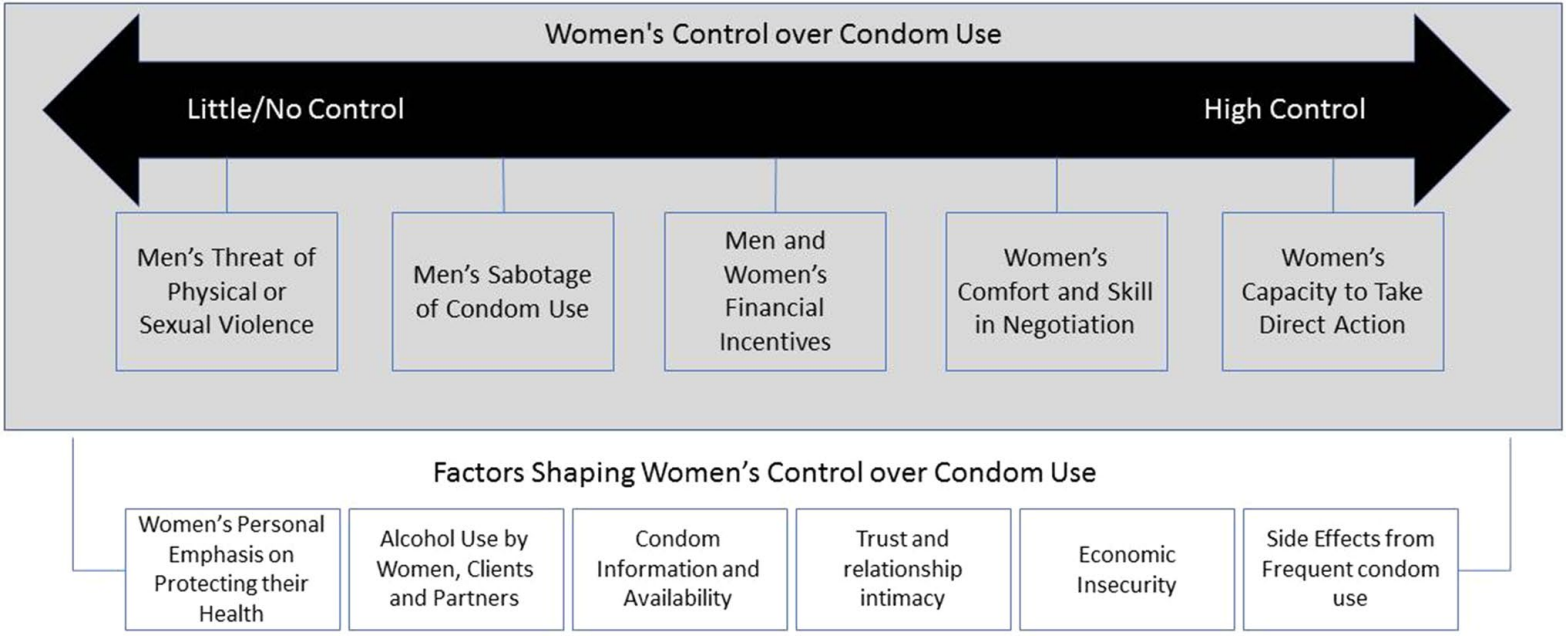
Conceptual model of factors influencing condom use among female entertainment workers in Cambodia

In identifying the factors impacting condom use for FEWs in Cambodia, a model emerged that contained elements along a continuum of women’s power, choice and control in using or negotiating condom use. Additionally, a number of factors were identified that had some influence over that continuum, shaping the degree to which women felt that they had some control over condom use in their sexual encounters (see Fig. 1). It is important to note that while questions were asked regarding condom use with different types of partners (e.g., clients, husbands or sweethearts), the identified themes spanned different relationship types, thus the results are not segmented by the type of sexual partner. Instead, we make an effort to identify which partners are being identified.

### Women’s Control Over Condom Use

These findings related to the degree to which women felt that they had power, choice and control are presented from little to no control, to high control.

#### Men’s Threat of Physical or Sexual Violence

This theme captured instances whereby women discussed how perceived or direct violence influenced the degree to which they felt they could advocate, or insist upon condom use. In eight of the 27 FGDs (29.6%), this theme was discussed. Several participants indicated that this violence, including physical or sexual assault or rape, made it most difficult for women to use condoms. One participant described her experiences: “Some may plead us or force us with physical violence or guns to convince us not to use” (Participant X, Phnom Penh Group 5). Another participant described her experiences of violence and how that resulted in condomless sex: “They forced me. They used violence… like sometimes they forced us to sleep with them and didn’t want to give use money. They didn’t use condoms” (Participant 2, Siem Reap Group 1).

While others were not directly threatened or had not experienced violence firsthand, they did state that in some instances, they had a generalized fear of the client, they had heard stories of violence, and they felt that fear inhibited their capacity to insist upon condom use.

#### Men’s Sabotage of Condom Use

In instances where women were able to successfully advocate for the use of condoms, they described times when men directly undermined them by either removing or breaking the condom, or the men lied about using one. This sabotage was discussed in eight of the 27 FGDs, representing all four geographic locales. In some cases, the women were aware of the man’s sabotage of the condom and could act accordingly. In other instances, women indicated that this happened without them knowing. One participant explained: “…they do not want to wear condoms, sometimes they have sex with us, and they unknowingly [to us] remove the condom, and that’s the problem” (Participant 2, Siem Reap Group 2).

When discussing what they thought motivated these actions, some women indicated that men did this to increase their own pleasure, which they felt entitled to, particularly clients. One participant described this: “They [clients] think that [condoms] reduces their sexual pleasure from 40 to 50%. And they think they pay for that [pleasure]” (Participant 2, Siem Reap Group 1).

Others discussed men breaking or compromising the condom deliberately to intentionally impregnate them. When asked to give an example, one participant described men’s sabotage of effective condom use: “Like when they want us to be pregnant so they break (make the hole to) the condom using needle” (Participant 6, Siem Reap Group 6.)

#### Men and Women’s Financial Incentives

Women described how condom use was often undermined by men providing some financial incentives for women to have unprotected sex. This theme was also discussed in seven of the 27 FGDs, in all four locations where FGDs were held. Financial incentives occurred in two ways. First, men would threaten not to pay for sex if the woman insisted upon the use of a condom. One participant described her experience with this:

He went into the room with me for such a long time and then he removed the condom. I refused and pushed him away then he refused to give me money. I didn’t agree and he said that if I didn’t, he’d call the police. I said ‘what an asshole’ but still he did not agree to give me the money. He forced me so hard until I ran downstairs (Participant 2, Banteay Meanchey Group 4).

Clients offering to pay more money for sex without a condom was another way that women’s decision-making capacity was shaped by financial incentives. When asked when it was most difficult to negotiate condom use, participants described this: “They say if we want more money, we must not use condoms” (Participant X, Phnom Penh Group 5). Several participants indicated that they used condoms consistently, except in instances where the client offered more money.

#### Women’s Comfort and Skills in Negotiation

In some instances, participants indicated that they felt comfortable negotiating condom use, with some stating they felt that they had a degree of control over the sexual encounter and even a right to insist upon condom use. Women discussed their comfort or skills in this negotiation process in 16 of the 27 FGDs (59.3%), representing all four provinces. When asked more specifically about how they negotiated this, women discussed a number of strategies including expressing a desire to avoid pregnancy, mentioning that they have some vaginal discharge or an STI, or encouraging the men to think about their own families. In discussing how she advocates for condom use with her sweetheart (regular client), one participant explained:

I negotiate with him about condom use and sometimes he doesn’t want to use but I explain to him, ‘I am afraid you would get infected from me. Don’t trust me; I have lots of clients and sometimes condoms are broken during sex.’ So to be safe for him and his wife, we should use condoms (Participant 1, Siem Reap Group 4).

#### Women’s Capacity to Take Direct Action

In addition to advocating for themselves verbally, women also indicated that there were a number of ways they took direct action, illustrating a high degree of power, choice and control. Specific strategies for direct action related to condom use were shared in 11 of the 27 FGDs, again representing all four provinces. In some cases, women stated that to ensure men used condoms, and did not sabotage them, they put the condom on directly. Other women refused to have sex without a condom. One participant stated unequivocally: “If my client does not use a condom, I refuse to have sex” (Participant 3, Phnom Penh Group 1). When asked if condom negotiation was difficult, another participant elaborated: “It’s not hard. It is a must. If they didn’t use a condom, I would step out from the room” (Participant 2, Battambang Group 2). Finally, some women indicated that they chose to have sex without a condom.

Some women choose not to use a condom with partners they trust. One participant, when asked if she used a condom with her sweetheart, responded: “never…he seems trustworthy, and I love him a lot…and he’s very loyal” (Participant 2, Siem Reap Group 5). Another, when asked why she did not wear a condom with her husband or sweetheart, simply stated: “They are my lovers. [I’m] using a condom with whom I don’t love only” (Participant 4, Siem Reap Group 4). Others indicated they did not use a condom for their or their partner’s pleasure.

### Factors Shaping Women’s Power, Choice, and Control

The factors shaping the degree to which women have power, choice or control are influenced by a number of factors, which are grouped into the below themes. Each one of the below themes was discussed in one or more FGDs in each of the four provinces.

#### Women’s Emphasis on Preserving Their Own Health

Several participants in 25 of the 27 FGDs (92.6%) contextualized the factors shaping their decision-making around condom use with an explicit emphasis on their desire to protect their health. Some women gave examples of ways that condom use was associated with health—e.g., avoidance of pregnancy and/or STIs, with little side effects: “When we use a condom, we don’t need to worry about transmission of diseases and using it won’t affect your health” (Participant 2, Battambang Group 1). Others emphasized a more global conceptualization of the importance of their health, and promoting it through condom use. As one participant put it, “we must love and protect ourselves by using condom” (Participant 6, Siem Reap Group 1).

#### Experiences with Side Effects

In 25.9% of the FGDs (seven out of 27), women indicated that their condom use (or lack thereof) was associated with the side effects from condoms experienced by either themselves or their partner. Side effects specific to women that were commonly cited included: itching, discharge, general inflammation, pain, burning, cervicitis (i.e., inflammation of the cervix) and irritation of the uterus. Two participants cited condoms as causing burning during urination or causing urine to become opaque; another indicated that it led to the development of a cauliflower or pealike mass in the uterus. One participant stated, “I trust my husband, but he bleeds every time we use condoms. And I am afraid of inflammation” (Participant 4, Phnom Penh Group 5). Another participant said, “I have been told that frequent use of condoms can harm my uterus” (Participant 2, Phnom Penh Group 5).

#### Alcohol Use

Participants indicated that the use of alcohol, both by the participant and her sexual partner, shaped the degree to which women felt that they had power, choice and/or control to use or negotiate the use of a condom. While alcohol was discussed more broadly across nearly every FGD, it was only discussed in relationship to condom use in five out of 27 groups (18.5%). Women indicated that when drinking alcohol, they may forget to ensure that the man is wearing a condom; it may decrease her resolve to insist upon a condom or it may lower her inhibitions, and she may have sex when a condom is not available. One participant described the risks of drinking and having sex without a condom: “Sometimes the women working at the club want to drink, and they become forgetful (some may not); they want to go out with the guests so the things they face are HIV and pregnancy” (Participant 3, Battambang Group 3).

When women were asked about the times that it is most difficult to persuade a man to wear a condom, they often discussed the challenges when their sexual partners were drinking. Men’s use of alcohol was often reported as being closely associated with them threatening violence, being difficult to negotiate with and generally unwilling to use a condom. One participant estimated that approximately 50% of FEWs experienced violence and attributed much of that to men who were drunk, explaining that “we refuse, they force us” (Participant 1, Banteay Meanchey, Group 4).

#### Condom Information and Availability

Across the interviews, participants were well-versed on where condoms were available, and where they could go for information about condom use and contraception more broadly. In 15 of the 27 FGDs (55.5%) women indicated that they have received information and condoms from medical providers and hospitals and clinics, community organizations and NGOs; motels often have condoms available as well. One participant explained where she got condoms: “We buy them…if not, the organization can distribute them” (Participants 8, Phnom Penh Group 5). Another participant followed-up stating: “The organization does that [distributes condoms] almost everyday…some motels also provide them” (Participant 3, Phnom Penh Group 5). Although women did not often cite it as a direct source of information about condoms, throughout the FGDs, they also talked about friends and family as informal sources of information regarding condoms.

#### Trust and Intimacy

Participants compared condom use decision-making with clients they didn’t know with regular clients sometimes called sweethearts and intimate partners. A participant’s trust of her sexual partner factored into her decision to insist upon or negotiate condom use. This trust as a driver of condom use (or of not using) was discussed in 16 of the 27 FGDs (59.3%). Participants often indicated that they would not necessarily use a condom with partners they trusted (excluding a desire to avoid pregnancy). This was most often the case in relationship to husbands or sweethearts (regular clients/boyfriends). In some instances, this meant that the women trusted that the men were not having sexual intercourse outside of their relationship. In other cases, women indicated that their husbands or sweethearts had other sexual partners, but they trusted the men were using protection in those instances. Additionally, a participant would also not insist upon or use a condom with a partner as a way to communicate to him that she trusted him. As one participant stated, “Because we found out we are HIV-negative, we have a close relationship with each other and we love each other, he doesn’t want to use condoms all the time” (Participant 4, Battambang Group 6).

Finally, women would often not wear a condom with her husband and sweetheart as a way to preserve his trust in her. In these instances, if the man is not aware that the woman has other sexual partners, she will often wear a condom with others, but not with her husband or sweetheart. One participant explained:

I never tell him I have sex with clients. I am afraid he will get angry and dump me so I need to hide from him…that’s why I use condoms only with my clients but with him I never use. I am afraid he suspects that I have more than one partner (Participant 2, Battambang Group 2).

#### Economic Insecurity

Undergirding, nearly every other theme in the model, was a woman’s economic insecurity. This theme was interwoven throughout all FGDs and was discussed both in direct and indirect relationship to condom use. The degree to which a woman felt as though she could insist upon or even negotiate condom use directly related to her financial stability. For women having sex with clients, economic insecurity related to their decision-making when clients offered more money for sexual intercourse without a condom and/or threatened to withhold payment.

Additionally, a woman’s financial standing also impacted her decision-making related to pregnancy and reproduction. Several FEWs indicated that they used condoms regularly to avoid pregnancy, as abortion was cited as being cost-prohibitive, as was the expense associated with raising a child. One married participant discussed her use of condoms given her limited capacity to financially support a child. She described the impact a pregnancy would have on her family:

If we have a lot of children, we don’t have enough time to take care them and enough money to support their living. So it is not only us who face the difficulty but also our child won’t get a bright future (Participant 5, Battambang Group 1).

## Discussion

This study found that FEWs experience a range of control over negotiating condom use with clients and partners in the post-100% condom use era in Cambodia. Participants reported times when they are able to take direct action and successfully insist on condom use, times when they agree to participate in condomless sex for money in the face of their economic insecurity and times when male clients sabotaged their attempts to negotiate condom use with tricks, verbal threats or threats of violence. These experiences are influenced by alcohol use by one or both parties, economic shocks, trust between partners and experiences with side effects. Our study is the only study to our knowledge that qualitatively assesses the challenges FEWs face to negotiating condom use without the monopoly environment of the original 100% CUP in Cambodia.

Studies from other countries have examined structural barriers to condom use. A study in China found waning success of their 100% condom use program and the need for a reinvigoration of condom use policies (Zou et al., 2011). A review of 24 qualitative studies of sex worker narratives globally underscored the role of third-party condom use enforcement such as venue managers, occupation safety measures or structural-level condom promotion programs on enforcing condom use (Goldenberg, Duff, & Krusi, 2015). The environment within venues has been shown to impact condom use, for example when managers and owner have rules about condom use (Morisky, Stein, Chiao, Ksobiech, & Malow, 2006). However, a study from Indonesia found that there was no increase on condom use despite sex workers’ perception of supportive policies (Safika, Levy, & Johnson, 2013).

Related research from other low-, middle- and high-income countries supports our findings in Cambodia on the factors that influence condom use among our population. The influence of alcohol consumption on condom use has been found among female sex workers in Kenya (Mbugua, Bukusi, Wagura, & Ngugi, 2017), Ghana (Onyango et al., 2015), Brazil (Szwarcwald et al., 2018), Korea (Jung, 2019) and Cambodia (Maher et al., 2011). The influence of economic insecurity or economic shocks such as a sick child on the decision to engage in condomless sex for higher pay has also been documented in Ghana (Onyango et al., 2015) and India (Reed et al., 2013). Other studies have shown that a multifaceted conceptualization of vulnerability that takes into account financial, structural and social vulnerability better explains female sex workers’ ability to negotiate condom use (Mahapatra, Bhattacharya, Atmavilas, & Saggurti, 2018; Ranebennur, Gaikwad, Ramesh, & Bhende, 2014).

Our study is one of only a few to examine the role of trust and intimacy played in FEWs condom use decision-making and negotiation. Few studies have examined the role of trust and intimacy in condom use between sex workers and clients. Relationship intimacy or trust has been documented as a key factor that influences condom use among sex workers and clients in Jamaica (Bailey & Figueroa, 2018) and Uganda (Duff et al., 2018). One study from Uganda found that sex workers develop a sense of trust with regular or repeat clients, which has a direct negative impact on consistent condom use (Duff et al., 2018). The studies that do exist document large differences in consistent condom use rates between casual and regular clients (Duff et al., 2018; Matovu & Ssebadduka, 2012).

Our study is unique in the findings that experiences of side effects by both men and women play an important role in condom use. There are limited studies that examine experiences with side effects relating to frequent condom use by female sex workers but some data on side effects were reported in an older study on lubricant use in Thailand (Rojanapithayakorn & Goedken, 1995) and general data on discomfort from condoms as it relates to decreased use is well-documented (Randolph, Pinkerton, Bogart, Cecil, & Abramson, 2007; Sanders et al., 2012). The frequency with which this was mentioned by participants in our data suggests that side effects from frequent condom use with and without lubrication may be an important barrier to condom use.

Some limitations should be considered when interpreting the findings of this study. Participants were selected using purposive sampling therefore our findings are not generalizable to the entire population of FEWs. It is possible that those that participated were more likely to be connected to ongoing HIV prevention programming and therefore might be more knowledgeable about condom use, have better access to condoms or may feel more confident in their ability to negotiate condom use. In this case, our findings would under represent the lived experiences of other less-connected FEWs.

Our focus group guide, although based on preliminary research and pilot testing, may have introduced bias into the discussions. In some cases, our focus group guide asks closed-ended questions to warm up the participants and then follows-up with open-ended questions. We structured the guide in this way because we found that speaking about condom use and condom negotiation in a group is difficult. In a focus group setting, the conversation is confidential but not anonymous which may limit the disclosure of personal information. Although this was not the intent of the sampling procedure, it is possible that participants knew each other from work or otherwise. This may have inhibited frank discussion about this topic although the quality of the data suggests that these dynamics did not hinder open discussions.

While some women in our study reported that they have developed strategies to negotiate condom use with clients or partners and reported reasonably good access to condoms, they continue to be vulnerable to condomless sex due to economic insecurity, men’s ability to sabotage condom use and clients threats of violence within a male-dominated society. Further research may focus on determining individual-level barriers to condom use including the role of side effects among clients and romantic partners, the role of trust between sex workers and regular clients and the role of individual venue policies in increasing consistent condom use. But past evidence suggests that structural-level changes are likely to be more impactful. Our findings support the development of national policies that re-invigorate the structural-level condom promotion programs (i.e., 100% CUP) in the post-brothel ban era or reversing the brothel ban entirely while also acknowledging that many other individual-level factors shape condom use.
